# 

**Published:** 1885-01

**Authors:** 


SUBSCRIPTION PRICE, $3.00 PER YEAR.

Whole Number, 301. JANUARY, 1885. Vol. L., Number 1.

LIBRARY
INDEXED
S.G.O.

### THE CHICAGO MEDICAL JOURNAL AND EXAMINER

EDITORS:

W. W. JAGGARD. HAROLD N. MOYER.

CHICAGO:

PUBLISHED BY THE CHICAGO MEDICAL PRESS ASSOCIATION,

193 WEST MADISON STREET.

CLARK & LONGLEY, PRINTERS, 163 AND 165 DEARBORN STREET, CHICAGO.

